# Optical coherence tomography of the retina combined with color Doppler ultrasound of the tibial nerve in the diagnosis of diabetic peripheral neuropathy

**DOI:** 10.3389/fendo.2022.938659

**Published:** 2022-10-21

**Authors:** Weimiao Chen, Xiaohong Wu, Shilin Li, Yan Zhang, Yinqiong Huang, Yong Zhuang, Xuefeng Bai, Xiaoyu Chen, Xiahong Lin

**Affiliations:** ^1^ Department of Clinical Nutrition, The Second Affiliated Hospital of Fujian Medical University, Quanzhou, China; ^2^ Department of Endocrinology, The Second Affiliated Hospital of Fujian Medical University, Quanzhou, China; ^3^ Department of Ultrasound, The Second Affiliated Hospital of Fujian Medical University, Quanzhou, China; ^4^ Department of Ophthalmology, The Second Affiliated Hospital of Fujian Medical University, Quanzhou, China; ^5^ Department of Endocrinology, The Seventh Affiliated Hospital of Sun Yat-sen University, Shenzhen, China; ^6^ Department of Geriatric Medicine, The Seventh Affiliated Hospital of Sun Yat-sen University, Shenzhen, China

**Keywords:** diabetes mellitus, peripheral neuropathy, optical correlation tomography, color doppler ultrasound, diagnosis

## Abstract

**Objective:**

To investigate the value of the retinal nerve fiber layer (RNFL) thickness in the optic disc and the cross-sectional area (CSA) of lower limb nerves in the diagnosis of diabetic peripheral neuropathy (DPN) separately and in combination.

**Methods:**

A total of 140 patients with type 2 diabetes were enrolled, including 51 patients with DPN (DPN group) and 89 patients without DPN (NDPN group). Clinical data and biochemical parameters were collected. Electromyography/evoked potential instrument was performed for nerve conduction study. Optical coherence tomography was performed to measure the RNFL thickness of the optic disc. Color Doppler ultrasound was performed to measure CSA of lower limb nerves.

**Results:**

The RNFL thickness was lower and the CSA of the tibial nerve (TN) in the DPN group was larger than that in the NDPN group. The album/urine creatinine ratio, diabetic retinopathy, and CSA of TN at 3 cm were positively correlated with DPN. The RNFL thickness in the superior quadrant of the optic disc was negatively correlated with DPN. For RNFL thickness to diagnose DPN, the area under the curve (AUC) of the superior quadrant was the largest, which was 0.723 (95% confidence interval [CI]: 0.645–0.805), and the best cutoff value was 127.5 μm (70.5% sensitivity, 72.1% specificity). For CSA of TN to diagnose DPN, the AUC of the distance of 5 cm was the largest, which was 0.660 (95% CI: 0.575–0.739), and the best cutoff value was 13.50 mm^2^ (82.0% sensitivity, 41.6% specificity). For the combined index, the AUC was greater than that of the above two indicators, which was 0.755 (95% CI: 0.664–0.846), and the best cutoff value was 0.376 (64.3% sensitivity, 83.0% specificity).

**Conclusions:**

Patients with DPN have a reduction of the RNFL thickness and an increase in the CSA of TN, and these two changes are related to DPN. The RNFL thickness of the optic disc and the CSA of TN can be used as diagnostic indicators of DPN, and the combination of the two indicators has a higher diagnostic value.

## 1 Introduction

The prevalence of diabetes is increasing year by year. According to the International Diabetes Federation, approximately 463 million adults (20–79 years old) are living with diabetes, and this will rise to 700 million by 2045 (1). Macrovascular and microvascular complications of diabetes are the main cause of morbidity and mortality of diabetes. Among them, diabetic peripheral neuropathy (DPN) is the most common diabetic microvascular complication. According to the statistics of the latest research, the prevalence of DPN for type 2 diabetes mellitus (T2DM) is 26.71% (2), and the prevalence of DPN for type 1 diabetes mellitus (T1DM) is 11% (3). The diagnosis of DPN is easily missed because of its hidden course and the characteristics of exclusive diagnosis. Approximately, 50% of DPN patients have no typical symptoms and need to be screened through physical and electrophysiological examinations. The consequences of DPN progression to late stage are severe, including pathological neuralgia, foot ulcers, Charcot’s joint, and even amputation (4, 5). DPN has a significant adverse impact on patients and society, but usually when DPN is diagnosed, its consequences are irreversible (6–8). In summary, the early screening and prevention of DPN are urgently needed.

The American Diabetes Association (ADA) (9) recommends that patients with T2DM and T1DM with a course of more than 5 years should be screened for complications every year. The screening for DPN includes symptom inquiry and five peripheral nerve tests (ankle reflex, vibration sensation, 10-g monofilament pressure sensation, superficial pain, and temperature sensation) (10). Nerve conduction study (NCS) is considered the most important DPN diagnosis method. Besides, quantitative assessment such as the Michigan neuropathy screening instrument is used for the screening of DPN. Corneal confocal microscopy, quantitative sensory testing, and skin biopsy technique are applied to diagnose small fibrous neuropathy. However, each of the above method has certain limitations. For example, some of them have poor repeatability and insufficient sensitivity and specificity, some are expensive and not accessible enough, and some are invasive methods that cannot easily be accepted by patients (11–15). Therefore, it is still necessary to explore convenient and efficient methods and strategies for screening and diagnosing DPN. From the perspective of anatomy, diabetes peripheral neuropathy can be divided into small fiber neuropathy, large fiber neuropathy, and mixed small and large fiber neuropathy (9). Retinal nerve fiber is a small unmyelinated nerve fiber, and the RNFL represents the axon of retinal ganglion cells. The tibial nerve (TN) is a large myelinated nerve fiber. Combining the assessment of large and small fiber neuropathy may improve the diagnostic efficiency for DPN. Previous studies of our team have shown that the use of optical coherence tomography (OCT) measuring the retinal nerve fiber layer (RNFL) thickness in the optic disc area has a value for clinical screening of DPN (16). Using OCT to measure the thickness of the RNFL is a method to assess small fiber neuropathy, and the detection method of large fiber neuropathy can be accomplished by color Doppler ultrasound (CDUS). In recent years, CDUS has been increasingly used in the evaluation of peripheral nerves. Many studies have proved that CDUS has broad prospects for application in the diagnosis of DPN (17). The above two examinations are both non-invasive, convenient, and inexpensive. In addition, they provided objective data indicators and relatively high repeatability. Combining the two examinations may improve the efficiency of early diagnosis of DPN. In this study, OCT was used to quantitatively measure the optic disc RNFL thickness, and CDUS was used to measure the cross-sectional area (CSA) of lower limb nerves. It is supposed to explore the diagnostic value of the combination of the two indicators and provide a new and efficient strategy for clinical screening and diagnosis of DPN.

## 2 Materials and methods

### 2.1 Subjects

Patients with T2DM who were hospitalized in the Department of Endocrinology of The Second Affiliated Hospital of Fujian Medical University (Quanzhou, China) from January 2018 to December 2020 were recruited. According to the inclusion and exclusion criteria, 140 patients were enrolled, including 51 patients with DPN (DPN group) and 89 patients without DPN (NDPN group). All the subjects were Chinese, with ages of 25–80 years. DPN was diagnosed according to the diagnostic criteria of the ADA position statement “confirmed DPN” (18). The inclusion criteria were as follows (1): patients with T2DM who met the diagnostic criteria of diabetes proposed by the World Health Organization in 1999 (2); corrected visual acuity of ≥4.6 (standard logarithmic visual acuity table); (3) diopter of ≤ ± 3.0 D; (4) binocular intraocular pressure range of ≤21 mm Hg (1 mm Hg = 0.133 kPa), no history of ocular hypertension; (5) no history of internal eye surgery, laser, or trauma; (6) no obvious abnormality in the anterior segment of the eye examined by a slit lamp; and (7) selection of the eye on the dominant hand side, and if it did not meet the standard, the other side was selected. The exclusion criteria were as follows: (1) fundus diseases caused by non-diabetes; (2) fundus examination with an obvious opacity of refractive media and diseases that could not be fixed; (3) acute or severe chronic complications of diabetes; (4) inflammatory or immune diseases, tumors, thyroid diseases, vitamin B deficiency, history of exposure to poison, or hereditary peripheral neuropathy; (5) neuropathy due to other causes like cerebral infarction, cerebral hemorrhage, Guillain–Barre syndrome cervical, lumbar lesions, and so on; (6) severe vascular diseases like lower limb arteriosclerosis obliterans, venous thrombosis in lower extremity, and so on; (7) history of severe trauma and surgery of the lower limbs; and (8) patients with severe cardiac, hepatic, and renal insufficiency. Flow chart of the study participants is shown in **Figure S1 in Supplementary Materials**.

### 2.2 Collection of clinical data

Clinical data and biochemical parameters were collected. Glycosylated hemoglobin (HbA1c) was determined by high-performance liquid chromatography (Model: D10, Bole Co., Ltd., USA). Urinary creatinine and urine microalbumin were detected by the automatic biochemistry analyzer (ARCHITECT c4000, Abbott Inc., USA). The estimated glomerular filtration rate and album/urine creatinine ratio (ACR) were calculated with **Formula S1** and **Formula S2** (**Supplementary Materials**).

### 2.3 Evaluation of diabetic peripheral neuropathy

#### 2.3.1 Clinical symptoms and peripheral nerve tests

A detailed inquiry and recording of clinical symptoms of DPN and five peripheral nerve tests (ankle reflex, vibration sensation, 10-g monofilament pressure sensation, superficial pain, and temperature sensation).

#### 2.3.2 Nerve conduction velocity

Nerve conduction velocity was detected by the same physician with an electromyography/evoked potential instrument (Keypoint 9033A07, Focus Company, Denmark), and the double-blind method was adopted. In a quiet room with a constant temperature of 25°C, the patient was in the supine position with limbs fully exposed and relaxed, and limb temperature was maintained at 32–36°C. It was performed according to the process recommended by the American Academy of Neurology and the American Association of Neuromuscular & Electrodiagnostic Medicine: (a) Measure the sensory conduction of the unilateral sural nerve (SN), median nerve, and ulnar nerve. (b) Measure the motor conduction and F wave of the unilateral common peroneal nerve, TN, median nerve, and ulnar nerve. Diagnostic criteria: It is judged to be DPN positive if there are abnormalities in two or more nerves, and one of which must be a lower limb nerve; otherwise, it is negative (19).

### 2.4 Ophthalmic examination

#### 2.4.1 Best corrected visual acuity and intraocular pressure

Best corrected visual acuity was measured with an autorefractor (Topcon KR 800, Topcon Medical Systems Inc., Japan) and corrected. Intraocular pressure was measured with a non-contact tonometer (Canon TX-20, Canon Inc., Japan).

#### 2.4.2 Slit lamp and front lens

A slit lamp (Topcon SL-3G, Topcon Medical Systems Inc., Japan) was used to observe the conjunctiva, cornea, anterior chamber, lens, and vitreous body in turn. The pupil was dilated with compound tropicamide eye drops, and then the fundus was examined with a slit lamp and Volk 90D front lens (Volk Inc., USA).

#### 2.4.3 Fundus photography

It was performed with a fundus camera (Canon CR-2, Canon Inc., Japan). The pupil was coaxial with the light, and the patients were told to move the eye in different directions. The physician then adjusted the focal length and definition of the image and selected the same ideal image as the object of study.

#### 2.4.4 Spectral domain optical coherence tomography

**Figure 1:**
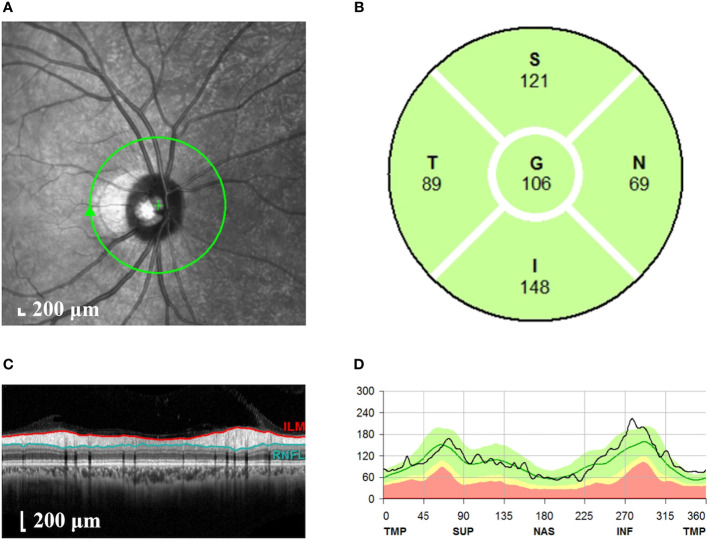
Measurement of the RNFL thickness of the optic disc. **(A)** Scanning image of the optic disc area. The green circle represents the scanning range. **(B)** Longitudinal section of the optic disc nerve fibers. The red line represents the internal limiting membrane; the blue line represents RNFL. **(C)** The thickness of RNFL in each quadrant of the optic disc. S, superior quadrant; N, nasal quadrant; I, inferior quadrant; T, temporal quadrant; G, overall average. **(D)** Pattern diagram of RNFL thickness. The abscissa indicates the quadrant, the ordinate indicates the thickness, the green line represents the normal value, and the black line represents the measured value. RNFL, retinal nerve fiber layer.

Spectral domain OCT (Spectralis^®^, Heidelberg Engineering Inc., Heidelberg, Germany) was used to scan the whole macular. The subject adopted a sitting position and placed their mandible on the jaw support at a suitable height. The subject was told to look at the green indicator light, and the lens was aimed at the pupil. The scanning centerline was adjusted to pass through the fovea center of the macular. The scanning mode was 768 × 496, and the scanning range was scanned with a macular fovea of 30°×25° volume. The scanning speed was 4,000 A/s, and the resolution was 5 μm longitudinally and 6 μm horizontally. A total of 61 B-scans were performed, and the mode of automatic real-time noise reduction was turned on to ensure that the quality of each scan was above 20 dB. For the RNFL measurement (**Figure 1**), we performed circular scanning of the optic disc with a diameter of 3.46 mm centered on the optic disc. The RNFL thickness was measured around the whole optic disc including four quadrants (superior, nasal, inferior, and temporal). Scanning was performed three times, and the clearest image with the strongest signal was selected and shown as a pattern diagram. Four-quadrant RNFL thicknesses and the overall average RNFL thickness were automatically analyzed using the analysis software supplied with the system. All of the above examinations were carried out by the same professionally trained ophthalmologist with the double-blind method.

### 2.5 Color Doppler ultrasound examination

**Figure 2:**
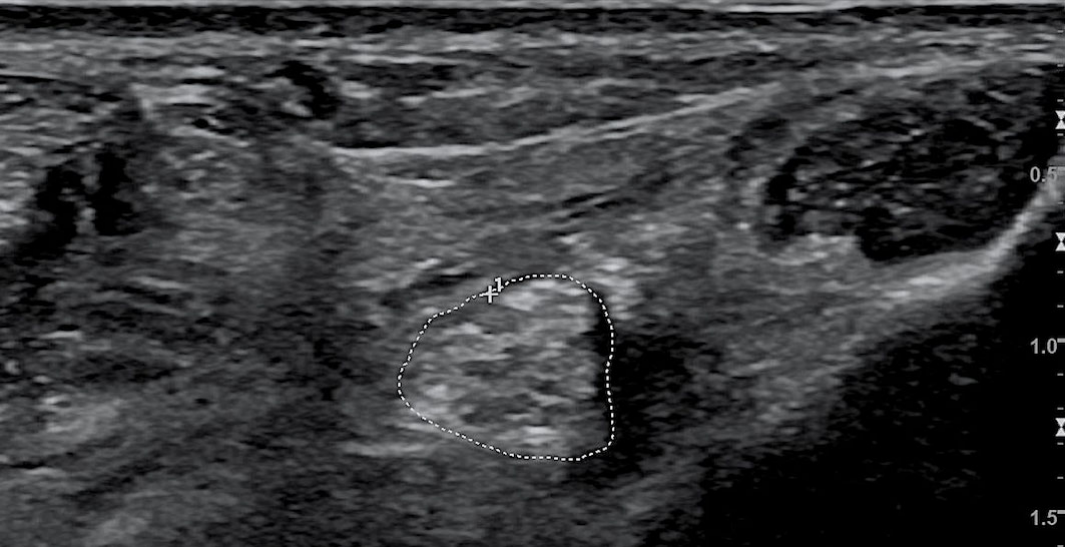
Color Doppler ultrasound image of the tibial nerve. The area circled by the dashed line is the CSA of the tibial nerve. CSA, cross-sectional area.

The examinations were performed by the same professionally trained sonographer with the double-blind method. CDUS (model: LOGIQ E9, GE Co., Ltd., USA) with linear array probe (model: ML6-15, GE Co., Ltd., USA) was used to detect nerves and measure the CSA of lower limb nerves. The probe frequency was 10–18 MHz, adjusted according to the depth of nerves. The subjects adopted a supine position in a quiet temperature-controlled room, with ankles positioned in slight plantar flexion and slightly rotated externally, cautioned not to move their feet or ankles during the CDUS examination. The sonographer detected the TN at the medial malleolus and 1, 3, and 5 cm to the medial malleolus and the SN at 10 and 20 cm to the heel. The nerve contour in the CDUS image based on the high echo of the nerve membrane was outlined by the sonographer, and the CSA of lower limb nerves were measured automatically (**Figure 2**).

### 2.6 Statistical analysis

SPSS 26.0 statistical software was used. The data were tested for normality, and those with a normal distribution were expressed as mean ± standard deviation, whereas those with a non-normal distribution were expressed as median (quartile intervals). For quantitative data with a normal distribution, Student’s t-test was used to test the significance of differences between the two groups. To measure data with a non-normal distribution, the Mann–Whitney U non-parametric test was used. The qualitative data were tested by the chi-square test. Binary logistic regression (LR backward) was used to analyze the correlation of clinical parameters, CSA of nerves, RNFL thickness, and DPN. The receiver operator characteristic (ROC) curve was drawn using MedCalc software (version 20.0.15, MedCalc Software Ltd., Belgium) to calculate the area under the curve (AUC). The CSA of nerves, RNFL thicknesses, and the combined index of the CSA of nerves and RNFL thicknesses in the diagnosis of DPN were compared. P < 0.05 was considered to indicate a statistically significant difference.

### 2.7 Compliance with ethics guidelines

All procedures followed were in accordance with the ethical standards of the responsible committee on human experimentation (institutional and national) and with the Helsinki Declaration of 1975, as revised in 2008. This study was approved by the Ethics Committee of The Second Affiliated Hospital of Fujian Medical University, Quanzhou, China. Informed consent was obtained from all patients for being included in the study.

## 3 Results

### 3.1 Clinical characteristics of the subjects

There were 51 cases in the DPN group, with an average age of 55.29 ± 12.44 years, and 89 cases in the NDPN group, with an average age of 51.12 ± 10.29 years. The duration of diabetes mellitus (DM) in the DPN group was longer than that in the NDPN group (9.57 [4.00, 13.00] vs. 5.64 [1.00, 8.50] years, p = 0.000). The systolic blood pressure (SBP) in the DPN group was higher than that in the NDPN group (130.80 ± 16.79 vs. 125.00 ± 11.79 mm Hg, p = 0.032). The blood urea nitrogen (BUN) in the DPN group was higher than that in the NDPN group (6.25 ± 3.74 vs. 5.01 ± 1.42 mmol/L, p = 0.033). The ACR in the DPN group was higher than that in the NDPN group (256.44 [10.08, 180.35] vs. 21.21 [6.10, 18.26] mg/g, p = 0.000). The diabetic retinopathy (DR) (%) in the DPN group was higher than that in the NDPN group (47.06% vs. 8.99%, p = 0.000) (**Table 1**).

**Table 1:** Clinical characteristics of the subjects in the DPN and NDPN groups.

Clinical characteristics	DPN group (n = 51)	NDPN group (n = 89)	p
Number of cases (male/female)	51 (36/15)	89 (50/39)	0.092
Age (year)	55.29 ± 12.44	51.12 ± 10.29	0.051
Duration of DM (year)	9.57 (4.00, 13.00)	5.64 (1.00, 8.50)	**0.000**
SBP (mm Hg)	130.80 ± 16.79	125.00 ± 11.79	**0.032**
DBP (mm Hg)	76.88 ± 10.7	76.73 ± 9.26	0.930
Height (m)	1.66 ± 0.08	1.65 ± 0.08	0.334
Weight (kg)	67.41 ± 13.49	66.43 ± 12.14	0.668
BMI (kg/m^2^)	24.48 ± 4.21	24.70 ± 3.56	0.787
FPG (mmol/L)	9.07 ± 3.44	9.12 ± 3.44	0.939
HbA1c [% (mmol/mol)]	9.4 ± 2.6 (79 ± 5)	8.8 ± 2.6 (73 ± 5)	0.749
ALT (mmol/L)	22.06 (14.00, 27.00)	30.11 (13.00, 33.00)	0.287
AST (mmol/L)	18.44 (14.00, 22.00)	21.82 (13.00, 24.00)	0.415
γ GT (mmol/L)	34.53 (17.00, 37.00)	34.27 (17.00, 37.50)	0.919
TG (mmol/L)	1.72 (1.02, 1.94)	2.20 (0.94, 2.37)	0.738
TC (mmol/L)	4.53 ± 1.17	5.07 ± 2.51	0.168
HDL (mmol/L)	1.15 ± 0.27	1.18 ± 0.40	0.650
LDL (mmol/L)	2.67 ± 1.03	2.74 ± 1.22	0.755
BUN (mmol/L)	6.25 ± 3.74	5.01 ± 1.42	**0.033**
SCr (mmol/L)	282.73 ± 1350.47	63.7 ± 16.60	0.262
SUA (μmol/L)	341.09 ± 98.55	332.27 ± 87.66	0.592
eGFR (ml/min/1.73m^2^)	99.67 ± 50.13	115.13 ± 28.23	0.051
ACR (mg/g)	256.44 (10.08, 180.35)	21.21 (6.10, 18.26)	**0.000**
DR (%)	47.06	8.99	**0.001**

DPN, diabetic peripheral neuropathy; NDPN, non–diabetic peripheral neuropathy; DM, diabetes mellitus; SBP, systolic blood pressure; DBP, diastolic blood pressure; BMI, body mass index; FBG, fasting blood glucose; HbA1c, glycosylated hemoglobin; DR, diabetic retinopathy; ALT, alanine transaminase; AST, aspartate transaminase; γ-GT, gamma glutamyl transpeptidase; TC, total cholesterol; TG, triglyceride; HDL, high-density lipoprotein; LDL, low-density lipoprotein; BUN, blood urea nitrogen; SCr, serum creatinine; SUA, serum uric acid; eGFR, estimated glomerular filtration rate; ACR, the album/urine creatinine ratio. The bolded and underlined data indicates the P<0.05.

### 3.2 Comparison of the retinal nerve fiber layer thickness in the optic disc area of the retina

There were significant differences in the overall average, superior quadrant, and inferior quadrant RNFL thicknesses. The RNFL thicknesses in the DPN group were lower than that in the NDPN group in the overall average (102.05 ± 12.91 vs. 106.94 ± 10.73 μm, p = 0.025), superior quadrant (119.28 ± 20.04 vs. 135.14 ± 17.72 μm, p = 0.000), and inferior quadrant (129.95 ± 18.12 vs. 140.98 ± 21.32 μm, p = 0.005). There were no significant differences in temporal quadrant or nasal quadrant RNFL thickness between the NDPN and DPN groups (**Table 2**).

**Table 2:** Comparison of RNFL thickness of the optic disc between the DPN and NDPN groups.

	DPN group (n = 51)	NDPN group (n = 89)	p
**Overall average (μm)**	102.05 ± 12.91	106.94 ± 10.73	0.025
**Temporal quadrant (μm)**	83.73 ± 18.91	80.20 ± 14.35	0.244
**Superior quadrant (μm)**	119.28 ± 20.04	135.14 ± 17.72	0.000
**Nasal quadrant (μm)**	77.12 ± 20.77	78.43 ± 18.37	0.718
**Inferior quadrant (μm)**	129.95 ± 18.12	140.98 ± 21.32	0.005

RNFL, retinal nerve fiber layer; DPN, diabetic peripheral neuropathy; NDPN, non–diabetic peripheral neuropathy. The bolded and underlined data indicates the P<0.05.

### 3.3 Comparison of the cross-sectional area of lower limb nerves

There were significant differences in the CSA of TN at 1, 3, and 5 cm to the medial malleolus. The CSA of TN in the DPN group was larger than that in the NDPN group at the distances of 1 cm (19.44 ± 6.63 vs. 17.31 ± 4.72 mm², p = 0.048), 3 cm (18.07 ± 5.75 vs. 15.81 ± 3.95mm², p = 0.016), and 5 cm (17.62 ± 5.01 vs. 15.01 ± 4.59 mm², p = 0.002) to the medial malleolus. There were no significant differences in the CSA of TN at the medial malleolus and the SN at 10 and 20 cm to heel between the NDPN and DPN groups (**Table 3**).

**Table 3:** Comparison of the CSA of lower limb nerves between the DPN and NDPN groups.

		DPN group (n = 51)	NDPN group (n = 89)	p
CSA of TN (mm^2^)	At 0 cm	20.22 ± 9.07	18.03 ± 5.64	0.128
	At 1 cm	19.44 ± 6.63	17.31 ± 4.72	**0.048**
	At 3 cm	18.07 ± 5.75	15.81 ± 3.95	**0.016**
	At 5 cm	17.62 ± 5.01	15.01 ± 4.59	**0.002**
CSA of SN (mm^2^)	At 20 cm	2.98 ± 1.76	2.79 ± 1.42	0.480
	At 10 cm	2.76 ± 1.21	2.66 ± 1.22	0.653

CSA, cross-sectional area; DPN, diabetic peripheral neuropathy; NDPN, non–diabetic peripheral neuropathy; TN, tibial nerve; SN, sural nerve. The bolded and underlined data indicates the P<0.05.

### 3.4 Correlation regression analysis of diabetic peripheral neuropathy

Binary logistic regression (LR backward) was used to analyze the correlation of clinical parameters, RNFL thickness of the optic disc area, CSA of lower limb nerves, and DPN. There were significant statistical differences in the duration of DM, SBP, BUN, ACR, DR, RNFL thickness in overall average, superior and inferior quadrant of the optic disc, and the CSA of TN at 1, 3, and 5 cm to the medial malleolus between the DPN group and the NDPN group. The above variables were taken as independent variables, and DPN was taken as a dependent variable. ACR (B = 0.008, p = 0.021), DR (B = 1.235, p = 0.014), and the CSA of TN at 3 cm from the medial malleolus (B = 0.157, p = 0.008) were positively correlated with DPN.The RNFL thickness in the superior quadrant of the optic disc (B = −0.037, p = 0.010) was negatively correlated with DPN (**Table 4**).

**Table 4:** The correlation of clinical parameters, CSA of TN, RNFL thickness, and DPN.

	B	Standard error	Wald	p	Exp(B)
**ACR**	0.008	0.003	5.315	0.021	1.008
**DR**	1.235	0.500	6.101	0.014	3.437
**Superior quadrant RNFL**	−0.037	0.014	6.641	0.010	0.964
**CSA of TN at 3 cm**	0.157	0.059	7.079	0.008	1.170
**Constant**	0.516	2.082	0.062	0.804	1.676

CSA, cross-sectional area; TN, tibial nerve; RNFL, retinal nerve fiber layer; DPN, diabetic peripheral neuropathy; ACR, album/urine creatinine ratio; DR, diabetic retinopathy. The bolded and underlined data indicates the P<0.05.

### 3.5 Diagnostic value of the cross-sectional area of the tibial nerve, optic disc retinal nerve fiber layer thickness, and the combined index for diabetic peripheral neuropathy

**Figure 3:**
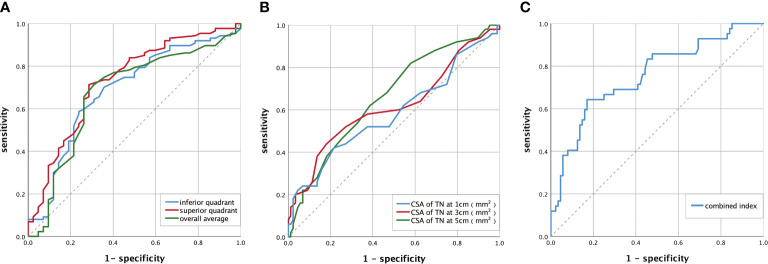
ROC curve of DPN diagnosed by RNFL thickness of the optic disc, the CSA of TN, and the combined index. **(A)** The ROC curve of DPN diagnosed by RNFL thickness of the optic disc. The AUC of the overall average was 0.675(95% CI: 0.587–0.755). The AUC of the superior quadrant was 0.723 (95% CI: 0.645–0.805). The AUC of the inferior quadrant was 0.686 (95% CI: 0.599–0.765). **(B)** The ROC curve of DPN diagnosed by CSA of TN. The AUC of the CSA of TN was 0.584 (95% CI: 0.497–0.667) at 1 cm, 0.617 (95% CI: 0.530–0.698) at 3 cm, and 0.660 (95% CI: 0.575–0.739) at 5 cm. **(C)** The ROC curve of DPN diagnosed by the combined index. The AUC of the combined index was 0.755 (95% CI: 0.664-0.846). AUC, area under the curve; ROC, receiver operator characteristic; DPN, diabetic peripheral neuropathy; CI, confidence interval; CSA, the cross-sectional area; TN, tibial nerve; RNFL, retinal nerve fiber layer. Combined index: the combined index of the CSA of TN at 3 cm and RNFL thickness in the superior quadrant. The calculation process is shown in **Supplementary Materials** and **Formula S3** (**Supplemental Data**).

The diagnostic values of the RNFL thickness in optic disc, the CSA of TN, and the combined index were evaluated by calculating the AUC of the ROC curve (**Figure 3**). A comparison of the diagnostic value of the indices is shown in **Table 5**, and the comparison of ROC curves is shown in **Table S1**. There was no significant difference in the AUC of the overall average, superior quadrant, or inferior quadrant RNFL thickness for DPN diagnosis. The AUC of the superior quadrant of RNFL thickness for diagnosing DPN was the largest, which was 0.723 (95% confidence interval [CI]: 0.645–0.805); sensitivity was 70.5%, and specificity was 72.1%; the best cutoff value was 127.50 μm. There was no significant difference in the AUC of the CSA of TN at 1, 3, and 5 cm from the medial malleolus for DPN diagnosis. The AUC of the CSA of TN at 5 cm for diagnosing DPN was the largest, which was 0.660 (95% CI: 0.575–0.739); sensitivity was 82.0%, and specificity was 41.6%; the best cutoff value was 13.50 mm^2^. The AUC of the combined index was greater than that of the above two indices, which was 0.755 (95% CI: 0.664–0.846); sensitivity was 64.3%, and specificity was 83.0%; the best cutoff value was 0.376.

**Table 5:** Comparison of RNFL thickness, CSA of TN, and the combined index in the diagnosis of DPN.

		AUC	Confidence interval	Cutoff value	Sensitivity	Specificity
RNFL thickness	Overall average	0.675	0.587–0.755	103.50	0.713	0.690
	Superior quadrant	0.723	0.645–0.805	127.50	0.705	0.721
	Inferior quadrant	0.686	0.599–0.765	138.50	0.591	0.762
CSA of TN	At 1 cm	0.584	0.497–0.667	20.50	0.420	0.784
	At 3 cm	0.617	0.530–0.698	18.50	0.440	0.809
	At 5 cm	0.660	0.575–0.739	13.50	0.820	0.416
Combined index		0.755	0.664–0.846	0.376	0.643	0.830

RNFL, retinal nerve fiber layer; CSA, cross-sectional area; TN, tibial nerve; DPN, diabetic peripheral neuropathy; AUC, area under the curve.

## 4 Discussion

This study suggests that patients with DPN have a reduction of the RNFL thickness and an increase in the CSA of TN, and these two changes are related to DPN. The RNFL thickness of the optic disc and the CSA of TN can be used as diagnostic indicators of DPN, and the combination of the two indicators has a higher diagnostic value. The AUC of the combined index was 0.755 (95% CI: 0.664–0.846); sensitivity was 64.3%, and specificity was 83.0%; the best cutoff value was 0.376.

DPN has a huge impact on the life of patients and brings a heavy burden to society. Early identification and treatment of DPN can relieve symptoms, reduce sequelae, and improve the quality of patients’ life. The ADA (9) recommend that patients with T2DM and T1DM with a course of more than 5 years should be screened for complications every year, including DPN. However, the proportion of routine DPN screening in clinical practice is still low (20). DPN is an exclusive diagnosis with insidious onset, and about half of DPN patients have no typical symptoms. A definitive diagnosis of DPN requires a complete and detailed symptom inquiry, a neurological examination, and an electromyography examination. There are limitations in all the existing screening and diagnosis methods. For example, some examination methods, such as the five peripheral nerve tests for DPN, have low repeatability. Twelve physicians assessed 24 patients with DM with physical features and voice disguised. The result showed that diagnosis from signs and symptoms was excessively variable among physicians (15). It may be because of the differences in the operation skills of the physicians and the subjective factors of the patients. Therefore, it is important to seek new ones.

DPN can be divided into small fiber neuropathy, large fiber neuropathy, or a mixture of large and small fiber neuropathy according to the types of nerve fibers involved (21). Some previous studies have suggested that small nerve fibers are abnormal and large nerve fibers are complete in the early stage of DPN (22). Unmyelinated small fibers are first affected during the DPN course, probably because they lack the protection provided by the myelin sheath (23, 24). Ziegler’s studies have shown that patients with early DPN had homogenous damage to both large and small nerve fibers (25). Therefore, the combined screening of large and small fiber neuropathy has a good theoretical basis as a strategy for screening DPN. Some studies have confirmed the feasibility of the DPN screening strategy by combining large and small nerves. Tesfaye used DPNCheck to evaluate large nerve fibers and Sudoscan to evaluate small nerve fibers for screening DPN. The prevalence of DPN was diagnosed at 51.5% (sensitivity 0.84, specificity 0.68) by DPNCheck, 38.2% (sensitivity 0.77, specificity 0.68) by Sudoscan, and 61.9% (sensitivity 0.93, specificity 0.53) by the combination of DPNCheck and Sudoscan (26). It improved the screening sensitivity and verified the feasibility of this strategy. A cross-sectional study of 145 patients with T2DM has shown that the combination of large fiber neuropathy assessed by NCS and small fiber neuropathy assessed by the thermal threshold test can identify the vast majority of patients with DPN, subclinical DPN, and clinical diagnosis of DPN (27). This study also inspires us that using the strategy of combining large and small nerves to screen DPN may be an effective way for the early detection of DPN. It may help in achieving early intervention of DPN, slowing down the disease progression and minimizing the harm. However, Sudoscan equipment is expensive and not easily accessible. The determination of the thermal threshold depends on the subjective feelings of the patient, and there are many influencing factors. In this study, we tried to find out two methods that are more commonly used and more accessible in clinical practice to detect large and small nerves, respectively, and combine them to diagnose DPN.

In recent years, a few studies have suggested that CDUS can be used for the assessment of peripheral neuropathy. It has the advantages of affordability, convenience, and non-invasiveness. The data indicate that ultrasound machines allowed real-time and centralized imaging of nerves and surrounding structures with high fidelity, which are able to quickly identify peripheral neuropathy (28–30). Besides, the patient has no discomfort or radiation exposure during the examination period. Diagnosis of neuropathy by CDUS is mainly based on the reliability of high resolution for measurement of large nerve fiber CSA (31, 32). In some previous studies, CDUS was used to detect CSA of peripheral nerves in patients with diabetes. It has been found that the CSA of the median nerve and TN in DPN patients is significantly higher than that in the non-DPN group. The degree of CSA increase is related to the severity of DPN. Our study showed that the CSA of TN was significantly larger in the DPN group than in the NDPN group at the distances of 1, 3, and 5 cm to the medial malleolus. The increase in nerve CSA is a manifestation of nerve swelling, and its main reasons include the increase in water content during the reductase conversion of glucose to sorbitol (33), the accumulation of microfibrillar material in the vicinity of perineurial cells, the increased diameter of collagen fibers in the endoneurium (34), the thickening of the vasculature and the combination of basement membrane and Schwann cells, etc. Using the CSA of nerve fibers measured by CDUS to diagnose DPN, sensitivity and specificity can reach 69.0%–80% and 70%–77%, respectively (35, 36). In a cross-sectional study, Riazi and his teammates used nerve ultrasound examination to detect DPN in patients. The result showed that the CSA of the posterior TN in the DPN group at the distances of 1, 3, and 5 cm to the medial malleolus was significantly larger compared with the control group. The CSA measured at 3 cm to the medial malleolus had an optimal threshold value for identification of DPN in their study, which was 19.01 mm^2^ (sensitivity 0.690, specificity 0.770) (36). In this study, the diagnostic value of CSA at 5 cm to the medial malleolus for the diagnosis of DPN is the greatest, and the cutting value is 13.50 mm² (sensitivity 0.820, specificity 0.416). There is no significant difference in the ROC curve of CSA of TN in diagnosing DPN at 1, 3, and 5 cm to the medial malleolus, so the result of this research is consistent with that of previous research. Based on the above, using CDUS to measure the CSA of nerve fibers may be an accessible and effective way to evaluate large nerves.

Previous views indicated that the retinal nerve was an extension of the central nerve and was closely related to central neuropathy. For example, retinal neurodegeneration often occurs in Alzheimer’s disease, multiple sclerosis, and Parkinson’s disease (37). In recent years, studies have found that retinal neuropathy is also closely related to peripheral neuropathy, and OCT has been introduced as a surrogate end point for evaluating retinal nerve fiber loss (38). Retinal nerve fiber is a small unmyelinated nerve fiber, and the RNFL represents the axon of retinal ganglion cells. It has been found that diabetic retinopathy is related to peripheral neuropathy; the RNFL thickness is related to the neuropathy disability score (NDS) of DPN patients. The RNFL thickness in the overall average, superior quadrant, and inferior quadrant of the optic disc in DPN patients with an NDS of ≥3 is significantly smaller than that in patients with diabetes but without DPN (39–41). These conclusions provide a reliable basis for RNFL thickness measured by OCT to diagnose DPN. Previous research by our team used OCT to measure the thickness of RNFL in the optic disc area and found that the thickness of RNFL was related to DPN and can be used as a diagnostic method for DPN. In this study, the result shows that RNFL thicknesses were significantly lower in the DPN group than that in the NDPN group in the overall average, superior quadrant, and inferior quadrant. However, there were no significant differences in temporal quadrant or nasal quadrant RNFL thickness between the NDPN and DPN groups. Studies have shown that there are significant differences in the shape of the four quadrants of the human posterior sclera sieve plate. The number and area of the ethmoidal foramen are significantly larger in the superior and inferior quadrants than in the nasal and temporal quadrants, so there are significantly more nerve fiber bundles in the superior or inferior quadrant than in the nasal or temporal quadrant (42). The decrease in nerve fiber bundle penetration indicates that the dynamic range of measurable RNFL thickness is very small, which may explain why there is no difference in RNFL thickness between the two groups in the temporal and nasal quadrants. The result of our previous study shows that the AUC of RNFL thickness in the inferior quadrant for diagnosing DPN was the largest, which was 0.755 (95% CI: 0.652–0.840, p < 0.001). The best cutoff value was 131 μm; sensitivity was 81.67%, and specificity was 68.97% (16). In this study, the AUC of the superior quadrant of RNFL thickness for diagnosing DPN is the largest, which is 0.723 (95% CI: 0.645–0.805). Sensitivity is 70.5%, and specificity is 72.1%; the best cutoff value is 127.5μm. There is no significant difference in the AUC of RNFL thickness in the overall average, superior quadrant, and inferior quadrant for the diagnosis of DPN. Therefore, the conclusion of this research is consistent with that of previous studies. Our study suggests that using OCT to measure RNFL thickness can be a feasible method to evaluate small fiber neuropathy. In addition, chronic diabetes complications must be regularly screened according to the global guidelines for the prevention and treatment of diabetes. Fundus examination of diabetes should include OCT to assess macular degeneration or edema. Early screening of DPN can be achieved while regularly evaluating fundus lesions in patients with diabetes. Therefore, it is a good choice to use OCT to evaluate small fiber neuropathy and screen DPN.

In this study, the RNFL thickness measured by OCT can reflect the lesions of small nerve fibers, whereas the CSA of the lower limb nerve measured by CDUS can reflect the lesions of large nerve fibers. Both examinations are non-invasive, are simple to operate, and have been widely used in clinical practice. Sensitivity/specificity of RNFL thickness, CSA, and the combined index to diagnose DPN was 70.5%/72.1%, 82.0%/41.6%, and 64.3%/83.0%, respectively. The AUC of the RNFL thickness, CSA, and the combined index were 0.723 (95% CI: 0.645–0.805), 0.660 (95% CI: 0.575–0.739), and 0.755 (95% CI: 0.664–0.846). The results show that the CSA of TN and RNFL thicknesses in the optic disc can be used as the diagnostic index of DPN, respectively. Furthermore, the DPN diagnostic value and specificity of the combination of CDUS and OCT is better than the CDUS or OCT alone. Therefore, the combination of neural CDUS and OCT in the diagnosis of DPN can be used as a new clinical strategy for the diagnosis of DPN. It is inspired by combining two methods detecting small and large fiber neuropathy could be a new strategy in DPN diagnosis. It may play an important role in clinical practice and benefit patients with DPN in the future. It is expected that DPN can be identified in an early stage and be intervened as soon as possible. Consequently, heavy stress caused by DPN on individuals and society could be released. In clinical practice, clinicians should make a reasonable choice of diagnostic methods according to the needs of the disease and the actual situation.

There are some limitations to this study. Because of the small sample size of this study, the subjects in the study were divided into the DPN group and the NDPN group, and the criteria for DPN was “confirmed DPN” according to ADA. In the consequent study, we will enroll more participants and divide the groups according to the definitions of minimal criteria for DPN (possible, probable, confirmed, and subclinical DPN) (18). Another limitation is that the RNFL thickness and the CSA of lower limb nerves are affected by many factors such as race, age, duration of diabetes, and the degree of retinal microangiopathy. Consequently, these findings must be verified by multicenter, large-sample, prospective observational studies.

## 5 Conclusions

Patients with DPN have a reduction of the RNFL thickness and an increase in the CSA of the TN, and these two changes are related to DPN. The RNFL thickness of the optic disc and the CSA of TN can be used as diagnostic indicators of DPN, and the combination of the two indicators has a higher diagnostic value.

## Data availability statement

The processed data required to reproduce these findings cannot be shared at this time as the data also forms part of an ongoing study. Requests to access the datasets should be directed to XL, 2413368792@qq.com.

## Ethics statement

The studies involving human participants were reviewed and approved by the Ethics Committee of the Second Affiliated Hospital of Fujian Medical University, Quanzhou, China. The patients/participants provided their written informed consent to participate in this study.

## Author contributions

XL designed the study. YaZ and S L performed Optical Coherence Tomography and Color Doppler Ultrasound examinations, respectively. WC, XW, XB, YoZ and X C collected the data. WC, XW, and YH performed data processing and data analysis. WC and XW wrote and edited the manuscript. All authors edited and approved the final manuscript, and they have full access to all the data in the study and take responsibility for the integrity and security of the data.

## Funding

This work was supported by the Fujian Provincial Health Technology Project, China (grant number 2020CXA044); the Natural science Foundation of Fujian Province, China (grant number 2020J01221); Key Young Talents Health Training Project of Fujian Province, China (grant number 2020GGA057).

## Acknowledgments

Many thanks to colleagues at the Department of Endocrinology at The Second Affiliated Hospital of Fujian Medical University (China) for providing comprehensive help to carry out the research.

## Conflict of interest

The authors declare that the research was conducted in the absence of any commercial or financial relationships that could be construed as a potential conflict of interest.

## Publisher’s note

All claims expressed in this article are solely those of the authors and do not necessarily represent those of their affiliated organizations, or those of the publisher, the editors and the reviewers. Any product that may be evaluated in this article, or claim that may be made by its manufacturer, is not guaranteed or endorsed by the publisher.
