# Supplementary material for: Optical coherence tomography of the retina combined with color Doppler ultrasound of the tibial nerve in the diagnosis of diabetic peripheral neuropathy
Source: Front Endocrinol (Lausanne). 2022 Oct 21;13:938659. doi: 10.3389/fendo.2022.938659 (PMC9634106; doi:10.3389/fendo.2022.938659)
Supplement: Supplementary file 1 [file DataSheet_1.docx]

**Supplementary Materials:**


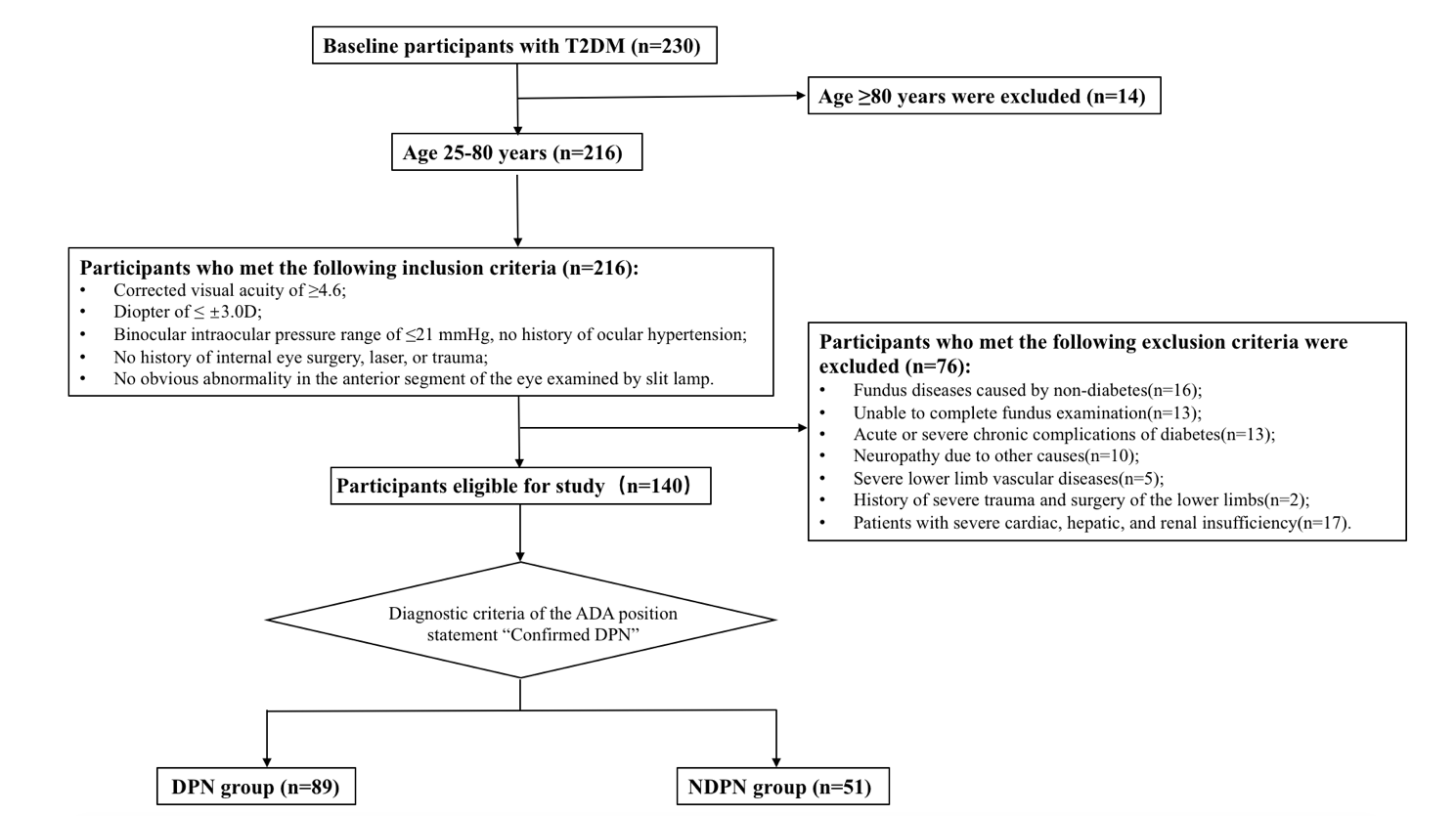


Figure S1: Flow chart of study participants

**Formula S1:**

$$\boldsymbol{eGFR(ml/min\cdot}\mathbf{1.73m}^{\boldsymbol{2}}\mathbf{)=}{\boldsymbol{186\times SCr}\left（ \mathbf{mg/dl} \right）}^{\boldsymbol{-1.154}}\boldsymbol{\times}{\mathbf{Age}\left( \mathbf{Y} \right)}^{\boldsymbol{-0.203}}\boldsymbol{\times(0.742 Female)}$$

eGFR: estimated glomerular filtration rate, calculated according to the simplified MDRD (Modification of Diet in Renal Disease) formula; SCr: serum creatinine.

**Formula S2:**

$$\boldsymbol{ACR(mg/g)=Alb(mg/L)\times1000}\mathbf{➗}\mathbf{UCr(umol/L)}\mathbf{➗}\boldsymbol{113.1\times1000}$$

ACR: Album/Urine Creatinine Ratio; Alb: **urinary** [microalbuminuria](link:microalbuminuria); UCr: urinary creatinine.

**Formula S3:**

$\boldsymbol{Combined index=}\frac{\boldsymbol{1}}{\mathbf{(}\boldsymbol{1+}\boldsymbol{e}^{\boldsymbol{-}\left( \boldsymbol{0.127\times A-0.046\times B+2.876} \right)}\mathbf{)}}$ 

A: CSA of TN nerve at 3cm; B: Superior quadrant RNFL thickness; CSA: cross-sectional area; RNFL: retinal nerve fiber layer.

Table S1: Comparison of ROC Curves

|  |  | AUC difference | Standard error | Z | P |
| --- | --- | --- | --- | --- | --- |
| CSA of TN at 1cm and at 3cm | | 0.033 | 0.044 | 0.749 | 0.454 |
| CSA of TN at 1cm and at 5cm | | 0.077 | 0.055 | 1.39 | 0.165 |
| CSA of TN at 3cm and at 5cm | | 0.044 | 0.040 | 1.096 | 0.273 |
| Overall average and Superior quadrant | | 0.055 | 0.048 | 1.143 | 0.253 |
| Overall average and Inferior quadrant | | 0.011 | 0.039 | 0.278 | 0.781 |
| Superior quadrant and Inferior quadrant | | 0.044 | 0.059 | 0.750 | 0.453 |
| CSA of TN at 5cm and Superior quadrant | | 0.063 | 0.069 | 0.913 | 0.361 |
| CSA of TN at 5cm and Combined index | | 0.069 | 0.028 | 2.423 | 0.015 |
| Superior quadrant and Combined index | | 0.006 | 0.048 | 0.120 | 0.905 |

ROC curve: receiver operator characteristic curve; RNFL: retinal nerve fiber layer; CSA: cross-sectional area; TN: tibial nerve.

Table S2: Regression correlation of RNFL thickness in superior quadrant, CSA of TN at 3cm and DPN

|  | B | Standard error | Wald | P | Exp(B) |
| --- | --- | --- | --- | --- | --- |
| Superior quadrant RNFL thickness | -0.046 | 0.012 | 13.44 | **0.000** | 0.956 |
| CSA of TN at 3cm | 0.127 | 0.047 | 7.254 | **0.007** | 1.136 |
| Constant | 2.876 | 1.701 | 2.860 | 0.091 | 17.74 |

RNFL: retinal nerve fiber layer; CSA: cross-sectional area. The two variables, CSA of TN at 3cm and Superior quadrant RNFL thickness, that were independently related to DPN, were selected to calculate a new variable, the combined index. Binary logistic regression analysis was used and the calculation process is shown in Formula S3.

Table S3: Abbreviation Index Table

| **Abbreviation** | **Full name** |
| --- | --- |
| AAN | American Academy of Neurology |
| AANEM | American Association of Neuromuscular & electrodiagnostic Medicine |
| ACR | Album/Urine Creatinine Ratio |
| ADA | American Diabetes Association |
| Alb | urine microalbumin |
| AUC | area under the curve |
| BCVA | best corrected visual acuity |
| BMI | body mass index |
| BUN | blood urea nitrogen |
| CCM | corneal confocal microscopy |
| CDUS | color Doppler ultrasonography |
| CI | confidence interval |
| CSA | cross section area |
| DM | diabetes mellitus |
| DPN | diabetic peripheral neuropathy |
| DR | diabetic retinopathy |
| ERG | electroretinogram |
| IDF | International Diabetes Federation |
| MNSI | Michigan neuropathy screening instrument |
| NCS | nerve conduction study |
| NCV | nerve conduction velocity |
| NDS | neuropathy disability score |
| OCT | optical coherence tomography |
| QST | quantitative sensory testing |
| RGCs | retinal ganglion cells |
| RNFL | retinal nerve fiber layer |
| ROC | receiver operating characteristic |
| SBP | systolic blood pressure |
| SN | sural nerve |
| SPSS | statistical product and service solutions |
| T1DM | type 1 diabetes mellitus |
| T2DM | type 2 diabetes mellitus |
| TN | tibial nerve |
| Ucr | urinary creatinine |
